# Analytical Performance of Two Flow Cytometry Systems for CD34⁺ Hematopoietic Stem Cell and CD3⁺ T-Cell Enumeration in Stem Cell Transplantation

**DOI:** 10.7759/cureus.99694

**Published:** 2025-12-20

**Authors:** Habsah Aziz, Suria Abdul Aziz

**Affiliations:** 1 Institute of Systems Biology, National University of Malaysia, Bangi, MYS; 2 Department of Pathology, Faculty of Medicine, Universiti Kebangsaan Malaysia, Kuala Lumpur, MYS; 3 Department of Diagnostic Laboratory Services, Hospital Canselor Tuanku Muhriz, Kuala Lumpur, MYS

**Keywords:** cd34⁺ hematopoietic stem cells, cd3⁺ t-cell, correlation, flow cytometry, precision, stem cell transplant

## Abstract

Background

Accurate enumeration of hematopoietic stem cells (HSC) and T-lymphocytes is essential for stem cell transplantation (SCT), as engraftment success depends on the infusion of adequate CD34⁺ and CD3⁺ cell doses. Flow cytometry is the gold standard for quantifying these subsets; however, analytical performance may vary between platforms, necessitating formal validation before clinical implementation. Therefore, this study aimed to evaluate the analytical precision of the DxFLEX flow cytometer and to assess its correlation and agreement with the established FACSCalibur reference method for CD34⁺ HSC and CD3⁺ T-cell enumeration.

Materials and methods

Analytical precision and method-comparison studies were conducted in accordance with Clinical and Laboratory Standards Institute (CLSI) EP15-A3 guidelines. Two levels of third-party quality control (QC) materials were used for CD34⁺ and CD3⁺ assays. Within-run precision was assessed using ten replicates per QC level (n =10), while between-run precision was evaluated across five days with three runs per day (n = 15). Mean values, standard deviations (SD), and coefficients of variation (CV) were compared with QC performance specifications. Method correlation was performed using 40 specimens following CLSI H57-A guidelines. Correlation coefficients and Bland-Altman analyses were used to assess agreement between the evaluated system (DxFLEX) and the reference analyzer (FACSCalibur).

Results

Both within-run and between-run precision for CD34⁺ and CD3⁺ measurements fell within acceptable QC limits, demonstrating stable assay performance. Correlation analyses showed excellent agreement between the two flow cytometry platforms, with correlation coefficients (r = 0.999 for CD34⁺ absolute counts and r = 1.00 for CD3⁺ absolute counts). Agreement between instruments was further supported by Bland-Altman analysis. For CD34⁺ measurements, the relative mean bias was −1.2% (limits of agreement: −33.4% to 31.1%) for percentage values and −0.479 (limits of agreement: −20.415 to 19.457) for absolute counts. For CD3⁺ measurements, the mean bias was −0.3% (limits of agreement: −3.5% to 2.9%) for percentage values and −0.358 (limits of agreement: −14.129 to 13.413) for absolute counts. These findings indicate strong concordance between the evaluated system and the reference platform, with minimal systematic differences and clinically acceptable limits of agreement.

Conclusion

The evaluated flow cytometry platform demonstrated excellent analytical performance for CD34⁺ and CD3⁺ enumeration, with precision and correlation results meeting clinical laboratory standards. Strong agreement with the reference method supports its suitability for routine use in HSC monitoring and SCT-related immunophenotyping.

## Introduction

Stem cell transplantation (SCT) is a cornerstone therapy for hematological malignancies and various hematological disorders. A critical determinant of successful engraftment is the number of viable hematopoietic stem and progenitor cells (HPCs), typically characterized as CD34⁺ cells, present in the infusion product [1]. Accurate enumeration of CD34⁺ cells in peripheral blood, apheresis products, bone marrow, or cord blood is therefore essential for SCT planning, mobilization timing, graft dose assessment, and quality control [2,3].

Flow cytometry has become the internationally accepted gold standard for CD34⁺ enumeration because it enables multiparametric analysis and provides both relative and absolute counts. The guidelines established by the International Society for Hematotherapy and Graft Engineering (ISHAGE) remain the benchmark for standardized and reproducible CD34⁺ enumeration [4].

In addition to CD34⁺ HSCs, the enumeration of CD3⁺ T-cells plays a critical role in various transplantation settings, including donor lymphocyte infusion, evaluation of graft composition, and monitoring immune reconstitution following transplantation. This requires a reliable and effective flow cytometric assay that differentiates viable intact lymphocytes from debris and dead cells, ensuring accurate quantification of the CD3⁺ cell population [5]. Furthermore, it has been noted that the composition of graft cells, specifically the ratio of CD34⁺ HSCs to CD3⁺ T-cells, can significantly impact the success of engraftment and the incidence of graft-versus-host disease (GVHD) [6].

In this study, we validated a contemporary flow cytometry platform using standardized QC materials and patient specimens to assess its analytical performance for both CD34⁺ HSC and CD3⁺ T-cell enumeration. By comparing its output with that of an established reference flow cytometer, we aimed to demonstrate whether the new system meets the rigorous requirements demanded in SCT-related immunophenotyping workflows.

## Materials and methods

Instrumentation, assay reagents, sample handling, and evaluation parameters

This evaluation study was conducted using two flow cytometry platforms: DxFLEX (Beckman Coulter Life Sciences, USA), which served as the test system, and the FACSCalibur™ (BD Biosciences, USA), which functioned as the established reference instrument. Multiple reagent kits were used for sample staining and cell enumeration. For hematopoietic progenitor cell analysis, the BCLS Stem-Kit™ reagent panel comprising CD45, CD34, and 7-AAD viability dye was used, while the BD™ Stem Cell Enumeration kit was included to support comparative assessments. For T-lymphocyte enumeration, a three-color antibody cocktail consisting of CD3 APC, CD45 FITC, and 7-AAD was applied to identify viable CD3⁺ T-cells and their corresponding lymphocyte and leukocyte subsets.

All samples used in this evaluation followed the collection, processing, transport, and storage requirements described in Clinical and Laboratory Standards Institute (CLSI) documents H3-A6 and H42-A2, ensuring integrity and suitability for flow cytometric analysis. The study utilized both quality control (QC) materials and patient specimens. CE-marked STRECK CD-CHEX CD34® Level 1 and Level 2 controls were run with the CD34⁺ and CD3+ assay to assess analytical performance across low and high expected values. Patient samples, including fresh peripheral blood and thawed cryopreserved apheresis specimens, were stained and processed following the manufacturer’s recommended protocols. To ensure optimal cell integrity and reduce pre-analytical variability, all samples were acquired on the flow cytometer within one hour of preparation.

Assay performance was assessed by evaluating both the percentage and absolute counts of viable CD34⁺ HPCs (CD34⁺/CD45⁺/7-AAD⁻), CD3⁺ T-cells (CD3⁺/CD45⁺/7-AAD⁻), lymphocyte subsets, and total CD45⁺ leukocytes. Marker selection was based on established immunophenotypic characteristics, where HPCs display CD34 and CD45 expression with low side scatter, and T-cells demonstrate strong CD3 and CD45 expression alongside lymphocyte scatter profiles. In all assays, 7-AAD viability dye was used to exclude dead or compromised cells, thereby improving accuracy and reducing enumeration bias.

Precision study

Precision of CD34⁺ and CD3⁺ enumeration was evaluated according to the principles outlined in CLSI EP15-A3, which defines precision as the closeness of agreement between independently obtained measurements under prescribed conditions. Precision encompasses both within-run precision, reflecting variability within a single analytical run, and between-run precision, which incorporates variability across multiple analytical runs over time. Total precision reflects the cumulative effect of both sources of measurement error.

Two levels of third-party QC materials (STRECK CD-CHEX CD34® Levels 1 and 2) were used for the precision evaluation. For within-run precision, each QC level was prepared, stained, and analyzed as ten independently processed aliquots (n = 10) within a single day by different operators, thereby incorporating both pre-analytical and analytical variability rather than instrument acquisition stability alone. For between-run precision, each QC level was independently prepared and analyzed three times per day over five consecutive days (n = 15), capturing day-to-day analytical variability.

Precision performance was expressed as the mean, standard deviation (SD), and coefficient of variation (CV). The coefficient of variation, defined as the ratio of the standard deviation to the mean, was used to describe the extent of dispersion relative to the magnitude of the measurement, with higher CV values indicating greater variability. Precision acceptability was evaluated against two criteria: (1) manufacturer-assigned QC performance specifications and (2) a laboratory-defined acceptance threshold of CV ≤ 15%, consistent with commonly applied criteria for flow cytometry quantification of low-frequency cell populations such as CD34⁺ HPC. The precision results were used to determine whether the evaluated DxFLEX flow cytometer demonstrated acceptable analytical stability and reproducibility for CD34⁺ and CD3⁺ enumeration.

Correlation study

A correlation study was performed to compare the analytical equivalency of the test system with the reference flow cytometer, following guidelines from CLSI H57-A, which describes comparability assessments between methods or instrument platforms. Correlation studies estimate systematic differences between methods and reveal whether constant or proportional error is present when real patient specimens are analyzed.

A total of 40 remnant clinical specimens (n = 40) were included in this study, comprising fresh peripheral blood samples and thawed cryopreserved apheresis specimens routinely encountered in stem cell transplantation (SCT) workflows. Each specimen was stained and prepared in parallel according to standardized protocols and analyzed sequentially on both flow cytometry systems within the same analytical window to minimize pre-analytical variability. Enumeration of CD34⁺ HPC followed the ISHAGE guidelines, employing a single-platform approach for the determination of both percentage and absolute CD34⁺ cell counts. CD3⁺ T-cell enumeration was performed using established immunophenotypic gating strategies based on CD3 and CD45 expression with viability exclusion.

Data analysis was performed using each instrument’s native acquisition and analysis software, with consistent gating logic applied across platforms in accordance with standardized assay protocols. CD34⁺ and CD3⁺ population plots generated from both systems were reviewed to assess linearity, measurement range, and the presence of outliers. For statistical comparison, the reference flow cytometer in routine clinical use was designated as the comparative method (x), while the evaluated system was designated as the test method (y).

Statistical evaluation included calculation of the Pearson correlation coefficient (r) to assess the strength of the linear relationship between methods. When r ≥ 0.99, simple linear regression was used to estimate systematic error across clinically relevant concentrations; when r < 0.975, alternative regression approaches such as Deming regression would be considered. Regression analysis provided slope and intercept values to evaluate proportional and constant bias between the two systems.

Agreement between the two methods was further examined using Bland-Altman analysis, which assesses mean bias and limits of agreement (LOA). Bland-Altman plots were generated by plotting the mean of paired measurements on the x-axis and the difference between methods on the y-axis. The mean difference (bias), 95% confidence intervals, and LOA (bias ± 1.96 standard deviations) were calculated. Both percentage values and absolute counts for CD34⁺ and CD3⁺ populations were included in the Bland-Altman analysis, allowing comprehensive assessment of agreement at parameters most relevant for clinical decision-making in SCT. These analyses were used to determine whether results obtained from the two systems could be considered interchangeable for routine clinical use.

## Results

Precision evaluation

The precision evaluation demonstrated that both CD34⁺ and CD3⁺ assays performed reliably across within-run and between-run assessments. For the CD34⁺ assay, all measured parameters, including CD34⁺ hematopoietic progenitor cells (HPCs) and CD45⁺ white blood cells (WBCs), were consistently within their established measuring ranges and met the acceptable QC specifications provided in the control package inserts. Within-run CVs for CD34⁺ Level 1 and Level 2 controls were 7.04% and 5.22%, respectively, while corresponding WBC CVs remained below 5%. Between-run precision also remained stable, with CVs of 7.16% for CD34⁺ Level 1 and 5.48% for CD34⁺ Level 2, and WBC CVs ranging from 5.97% to 6.38%. All results were within manufacturer-defined acceptable limits, confirming consistent instrument and assay performance (Table 1).

**Table 1:** Precision Analysis of CD34⁺ Enumeration: Within-Run and Between-Run Results

QC CD-Chex CD34®	Parameter (cells/µL)	Mean	SD	CV (%)	Measuring Range	± 2SD	Status
WITHIN - RUN							
Level 1	CD34^+^	11.88	0.84	7.04	8.0 – 16.0	≤ 4.00	PASS
	WBC (× 10^3 ^/µL)	6.48	0.14	2.16	5.6 – 7.6	≤ 1.00	PASS
Level 2	CD34^+^	31.88	1.66	5.22	24.9 – 38.9	≤ 7.00	PASS
	WBC (× 10^3^/µL)	6.18	0.25	4.02	5.6 – 7.6	≤ 1.00	PASS
BETWEEN – RUN							
Level 1	CD34^+^	11.53	0.83	7.16	8.0 – 16.0	≤ 4.00	PASS
	WBC (× 10^3^/µL)	6.30	0.38	5.97	5.6 – 7.6	≤ 1.00	PASS
Level 2	CD34^+^	30.97	1.70	5.48	24.9 – 38.9	≤ 7.00	PASS
	WBC (× 10^3^/µL)	6.04	0.39	6.38	5.6 – 7.6	≤ 1.00	PASS

Similarly, the CD3⁺ assay demonstrated strong precision across both QC levels. Within-run CVs ranged from 3.08% to 6.26% for CD3⁺ and lymphocyte counts, while WBC CVs remained below 5.09%. Between-run CVs were also acceptable, with CD3⁺ values of 8.90% and 12.31% for Level 1 and Level 2, respectively, and lymphocyte CVs between 9.08% and 12.41%. WBC CVs for both levels remained within the allowable ±2SD performance criteria. Across all QC runs, most precision data points fell well within the 2SD control range, demonstrating consistent day-to-day instrument stability and reproducibility (Table 2).

**Table 2:** Precision Analysis of CD3⁺ Enumeration: Within-Run and Between-Run Results

QC Streck ChexPlus BC	Parameter (cells/ µL)	Mean	SD	CV (%)	Measuring Range	± 2SD	CV (%)	Status
WITHIN RUN								
Level 1	CD3^+^	1925.53	59.33	3.08	1538 – 2738		≤ 14.0	PASS
	Lymphocyte	2483.45	77.86	3.14	1954 – 3274		≤ 12.0	PASS
	WBC (× 10^3^/µL)	6.84	0.16	2.41	5.6 – 7.6	≤ 1.00		PASS
Level 2	CD3^+^	952.44	57.11	6.00	684 – 1564		≤ 19.0	PASS
	Lymphocyte	1596.61	100.02	6.26	1307 – 2228		≤ 13.0	PASS
	WBC (× 10^3^/µL)	6.76	0.34	5.09	5.6 – 7.6	≤ 1.00		PASS
BETWEEN RUN								
Level 1	CD3^+^	1953.25	173.91	8.90	1538 – 2738		≤ 14.0	PASS
	Lymphocyte	2515.61	228.43	9.08	1954 – 3274		≤ 12.0	PASS
	WBC (× 10^3^/µL)	6.84	0.54	7.87	5.6 – 7.6	≤ 1.00		PASS
Level 2	CD3^+^	996.38	122.65	12.31	684 – 1564		≤ 19.0	PASS
	Lymphocyte	1667.26	206.96	12.41	1307 – 2228		≤ 13.0	PASS
	WBC (× 10^3^/µL)	6.99	0.74	10.53	5.6 – 7.6	≤ 1.00		PASS

Collectively, these results indicate that the evaluated flow cytometry system provides consistent and reliable measurements across repeated analyses, supporting its suitability for clinical CD34⁺ and CD3⁺ enumeration.

Correlation and agreement study

**Figure 1:**
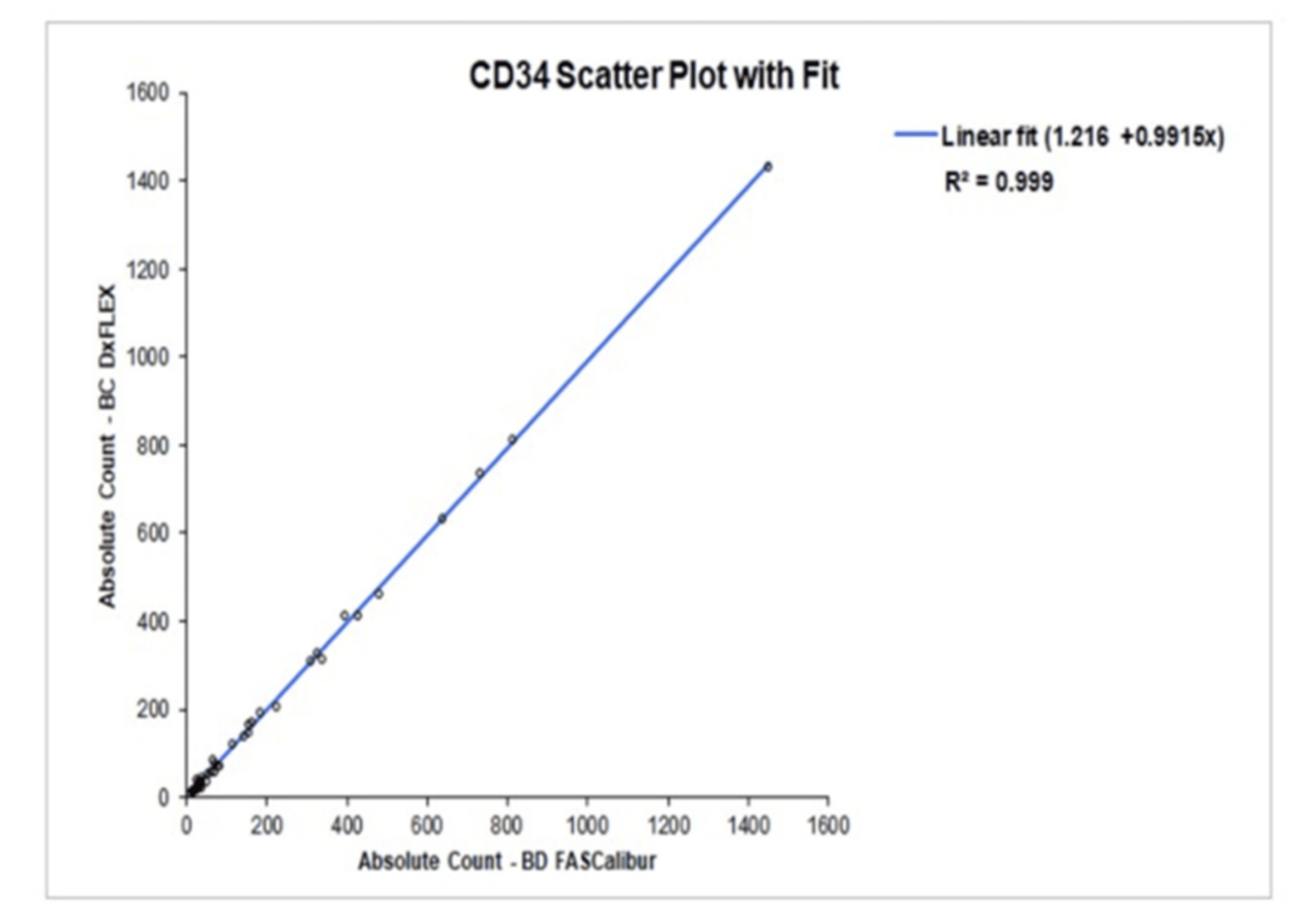
Linear regression analysis demonstrating correlation of CD34⁺ absolute counts between the evaluated flow cytometry system and the reference flow cytometry method.

**Figure 2:**
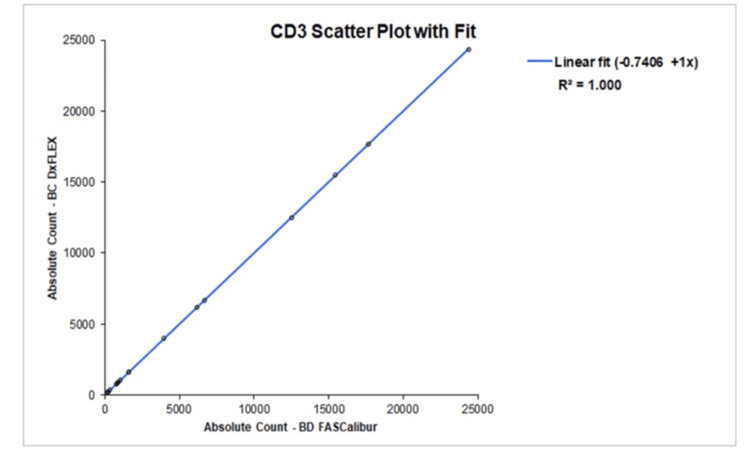
Linear regression illustrating excellent concordance in CD3⁺ absolute counts between the evaluated cytometry platform and the reference method.

Correlation analysis demonstrated excellent agreement between the evaluated flow cytometer and the reference system. For CD34⁺ absolute counts, the correlation coefficient (r) reached values consistent with near-perfect linearity, confirming strong equivalence between the two analytical platforms (Figure 1). CD3⁺ absolute counts demonstrated a similarly robust correlation, with r values meeting the threshold indicative of highly reliable inter-instrument comparability (Figure 2). These findings confirm that both platforms quantify CD34⁺ HPCs and CD3⁺ T-cells in a closely comparable manner.

**Figure 3:**
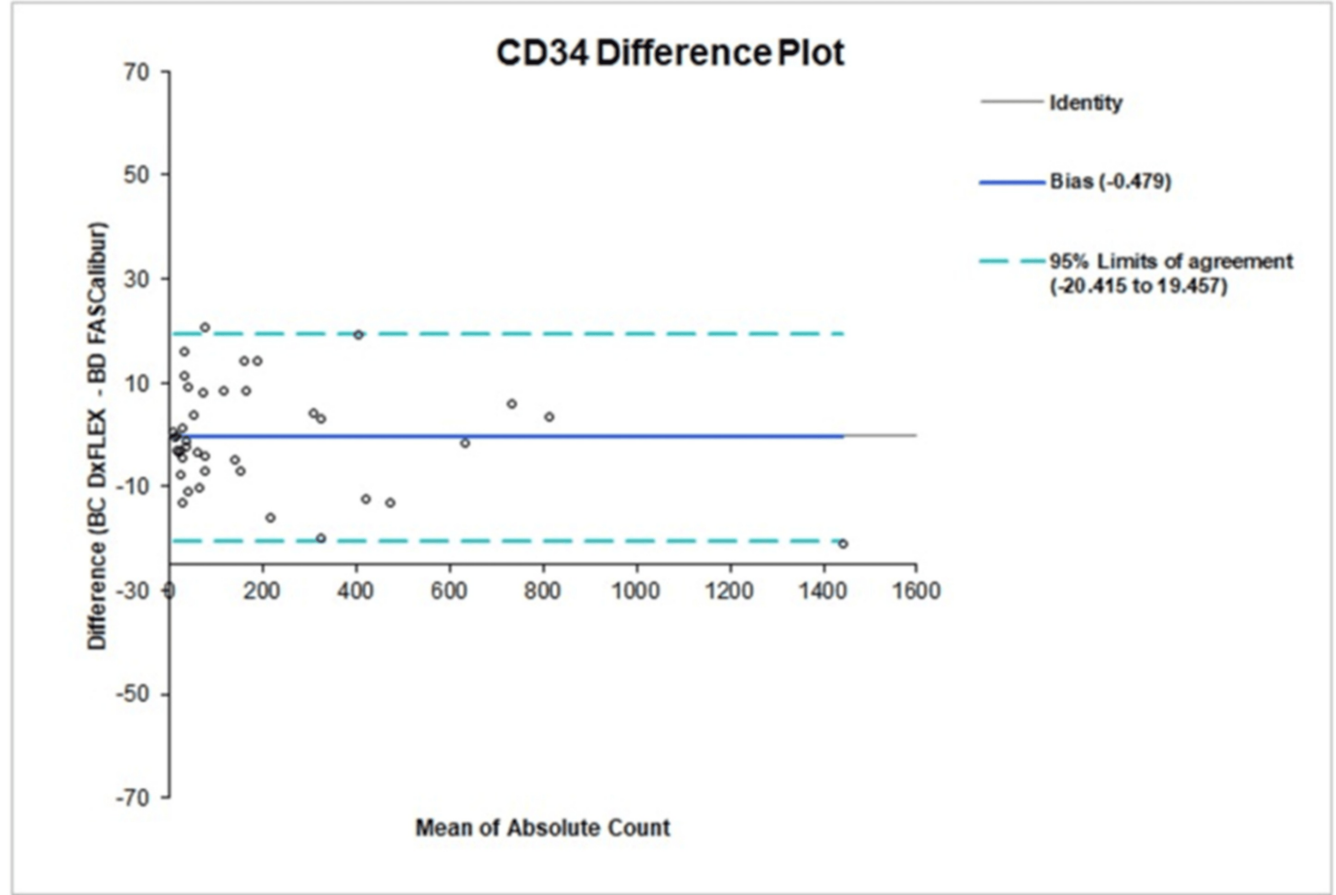
Bland-Altman analysis of CD34⁺ absolute counts comparing the evaluated flow cytometry system with the reference method, showing mean bias and limits of agreement.

**Figure 4:**
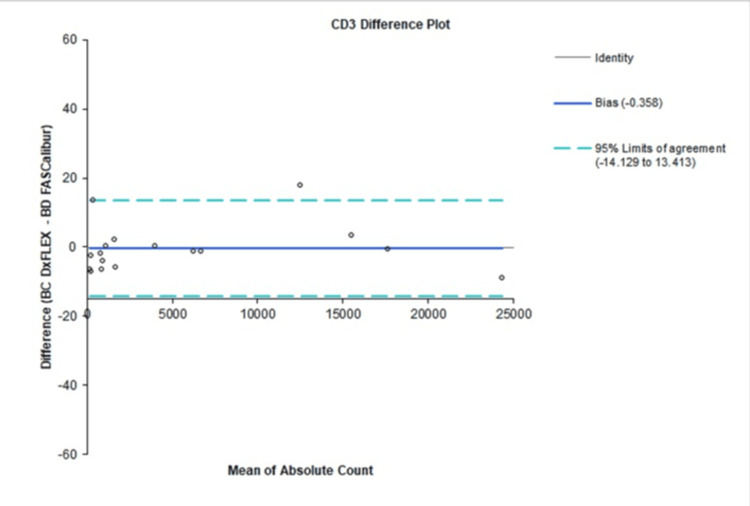
Bland-Altman plot showing bias and limits of agreement for CD3⁺ absolute counts between the evaluated system and the reference platform.

Agreement between instruments was further assessed using Bland-Altman analysis. For CD34⁺ measurements, the relative mean bias was −1.2% (LOA: −33.4% to 31.1%) for percentage values and −0.479 (−20.415 to 19.457) for absolute counts (Figure 3). For CD3⁺ measurements, the mean bias was −0.3% (LOA: −3.5% to 2.9%) for percentage values and −0.358 (−14.129 to 13.413) for absolute counts (Figure 4). These values demonstrate that differences between methods were small, symmetrically distributed, and lacked any meaningful proportional or constant error.

Overall, the Bland-Altman findings confirm high concordance between the two instruments for both CD34⁺ and CD3⁺ cell counts. The narrow LOA and minimal bias observed for all analytes suggest that measurements from the evaluated system are clinically interchangeable with those obtained from the reference platform. Taken together, these precision, correlation, and agreement results demonstrate excellent analytical comparability and support the suitability of the evaluated flow cytometer for routine CD34⁺ and CD3⁺ enumeration in SCT workflows.

## Discussion

Flow cytometers require rigorous analytical performance evaluation, including precision, reproducibility, assay stability, and comparability to reference methods, before adoption in regulated clinical settings like SCT [7]. In this study, both the CD34⁺ hematopoietic stem cell (HSC) assay and the CD3⁺ T-lymphocyte assay demonstrated strong analytical performance, confirming the suitability of the evaluated system for clinical laboratory use.

The precision results showed that both within-run and between-run variability for CD34⁺ and CD3⁺ measurements fell within acceptable QC limits. These findings indicate stable assay performance across repeated measurements and over multiple days, a requirement for consistent SCT-related testing. Although minor operator-related variability during sample preparation was observed, this did not impact overall assay reliability. Operator effects are well documented in flow cytometry, particularly in assays requiring multiple pipetting and staining steps, and are considered an expected component of biological and technical variability [7,8].

Method comparison against an established reference platform demonstrated excellent concordance. These coefficients indicate how well measurements from one method correspond to those of the other, with values closer to 1 (or -1) showing strong linear correlation [9,10]. Correlation coefficients (r) reached 1.00 for both CD34⁺ and CD3⁺ absolute counts, indicating nearly perfect linearity between instruments. According to commonly accepted method-comparison criteria, correlation coefficients ≥ 0.99 are indicative of strong agreement and allow reliable estimation of systematic error. When correlation falls below 0.975, alternative statistical approaches such as Deming regression are recommended; however, such measures were unnecessary in the present study due to the consistently high correlation observed [11].

Bland-Altman analysis further supported instrument comparability [10]. For CD34⁺ measurements, the relative mean bias was −1.2% for percentages and −0.479 for absolute counts, with LOA that remained clinically acceptable. Similarly, CD3⁺ assays demonstrated minimal bias, with −0.3% for percentage values and −0.358 for absolute counts. These small differences fall well within the acceptable threshold for biological assays and are comparable to previously reported inter-instrument variability in flow cytometry-based cell enumeration. Importantly, the narrow LOA indicates that the evaluated system can be used interchangeably with the reference platform in clinical workflows.

The minimal biases observed for both CD34⁺ and CD3⁺ measurements likely reflect normal assay-related variation rather than true methodological disparities. Contributing factors may include pre-analytical variables, such as sample handling times, temperature fluctuations, and pipetting accuracy. Differences in gating strategy, cytometer flow rate, signal processing, and threshold settings may also introduce small shifts in population quantification. Additionally, biological factors such as cell debris, aggregates, or the presence of dead or dying cells, particularly in thawed apheresis samples, may influence apparent CD34⁺ or CD3⁺ frequencies if not adequately excluded. The incorporation of viability dyes such as 7-AAD helps mitigate these effects but cannot eliminate them entirely.

Overall, the precision, correlation, and agreement results indicate that the evaluated flow cytometry system provides robust analytical performance for CD34⁺ and CD3⁺ enumeration. Its ability to deliver reproducible results across multiple runs and close agreement with a well-established reference method supports its integration into SCT-related immunophenotyping workflows. The flexibility of the platform, compatibility with standardized reagents, and capacity to support multicolor assays further enhance its suitability for routine clinical use. Continued monitoring of instrument performance through routine QC procedures and periodic cross-platform comparisons remains essential to ensure sustained assay accuracy.

This study has several limitations that should be acknowledged. First, although the sample size was adequate for method comparison under CLSI H57-A guidelines, the cohort did not encompass the full biological variability seen across other clinically relevant sample types, such as cord blood, bone marrow, or mobilized apheresis products, which may limit generalizability. Second, the analytical comparison was performed exclusively against the BD FACSCalibur™ flow cytometer, a well-established but older-generation platform. The inclusion of multiple contemporary cytometers would provide a broader assessment of analytical equivalence. Third, the evaluation focused on precision and method agreement under controlled laboratory conditions, without assessment of long-term performance characteristics. Future studies incorporating larger and more diverse sample sets, comparisons with additional modern platforms, and prospective longitudinal monitoring would further strengthen the validation of the evaluated system for widespread clinical application.

Despite these limitations, a key strength of this study lies in its rigorous, standards-based validation framework, conducted in accordance with CLSI EP15-A3 and H57-A guidelines using commercial third-party controls. Importantly, this work provides a practical validation roadmap for clinical laboratories transitioning from legacy flow cytometry platforms to newer systems in SCT workflows.

## Conclusions

This evaluation demonstrated that the tested flow cytometry system provides reliable, precise, and clinically comparable measurements for both CD34⁺ HPC and CD3⁺ T-lymphocytes. Precision analysis showed that within-run and between-run variability consistently fell within acceptable QC limits, confirming stable analytical performance across different testing conditions. Method comparison with the reference flow cytometer revealed excellent linear correlation for absolute CD34⁺ and CD3⁺ counts, supported by Bland-Altman analyses that demonstrated minimal bias and narrow LOA for both percentage and absolute values. These outcomes indicate that the two instruments generate highly concordant results, with no clinically meaningful differences that would affect decision-making in SCT workflows. Overall, the evaluated DxFLEX flow cytometry platform meets the analytical requirements for routine CD34⁺ and CD3⁺ enumeration and can be confidently implemented as a suitable alternative to the established reference system in clinical laboratory practice.

## References

[1] Vignon C, Lachot S, Foucault A (2020). Reactive oxygen species levels differentiate CD34(+) human progenitors based on CD38 expression. Cytometry B Clin Cytom.

[2] Haussmann K, Streitz M, Takvorian A (2022). Widely applicable, extended flow cytometric stem cell enumeration panel for quality control of advanced cellular products. Sci Rep.

[3] Murugesan M, Nair CK, Nayanar SK, Pentapati KC (2019). Flow cytometric enumeration of CD34+ hematopoietic stem cells: A comparison between single- versus dual-platform methodology using the International Society of Hematotherapy and Graft Engineering protocol. Asian J Transfus Sci.

[4] Sutherland DR, Anderson L, Keeney M, Nayar R, Chin-Yee I (1996). The ISHAGE guidelines for CD34+ cell determination by flow cytometry. International Society of Hematotherapy and Graft Engineering. J Hematother.

[5] Svenberg P, Wang T, Uhlin M (2019). The importance of graft cell composition in outcome after allogeneic stem cell transplantation in patients with malignant disease. Clin Transplant.

[6] Saad A, Lamb L, Wang T (2019). Impact of T cell dose on outcome of T cell-replete HLA-matched allogeneic peripheral blood stem cell transplantation. Biol Blood Marrow Transplant.

[7] Maecker HT, McCoy JP Jr, Amos M (2010). A model for harmonizing flow cytometry in clinical trials. Nat Immunol.

[8] Oldaker TA, Wallace PK, Barnett D (2016). Flow cytometry quality requirements for monitoring of minimal disease in plasma cell myeloma. Cytometry B Clin Cytom.

[9] Wiśniewska A, Stańczyk J, Demkow U, Stelmaszczyk-Emmel A (2024). Peripheral blood lymphocyte immunophenotyping (TBNK) - a comparison of BD FACSCanto II and BD FACSLyric flow cytometry analysers. Cent Eur J Immunol.

[10] Giavarina D (2015). Understanding Bland Altman analysis. Biochem Med (Zagreb).

[11] Linnet K (1993). Evaluation of regression procedures for methods comparison studies. Clin Chem.

